# Cardiovascular Involvement in Thyrotoxicosis Resulting in Heart Failure: The Risk Factors and Hemodynamic Implications

**DOI:** 10.7759/cureus.21213

**Published:** 2022-01-13

**Authors:** Ciri C Raguthu, Harini Gajjela, Iljena Kela, Chandra L Kakarala, Mohammad Hassan, Rishab Belavadi, Sri Vallabh Reddy Gudigopuram, Ibrahim Sange

**Affiliations:** 1 Research, Tianjin Medical University, Tianjin, CHN; 2 Research, Our Lady of Fatima University College of Medicine, Valenzuela, PHL; 3 Family Medicine, Jagiellonian University Medical College, Kraków, POL; 4 Internal Medicine, Jawaharlal Institute of Postgraduate Medical Education and Research (JIPMER), Pondicherry, IND; 5 Internal Medicine, Mohi-ud-Din Islamic Medical College, Mirpur, PAK; 6 Surgery, Jawaharlal Institute of Postgraduate Medical Education and Research (JIPMER), Pondicherry, IND; 7 Research, Our Lady of Fatima University, Hyderabad, IND; 8 Research, K. J. Somaiya Medical College, Mumbai, IND

**Keywords:** thyroid, hyperthyroidism, cardiovascular complications, heart failure, thyrotoxicosis

## Abstract

Thyrotoxicosis is a clinical syndrome with persistently elevated concentrations of free triiodothyronine, free thyroxine, or both, which correlates with an increased thyroid metabolic function. This article has discussed the direct effect of increased thyroid hormone on the heart, as the thyroid hormone physiologically exhibits a close harmony with hormones of the cardiovascular system. This action can lead to disturbances in hemodynamic stability, exacerbating the possibility of developing complications such as heart failure and life-threatening arrhythmias. This article has also explored the multifaceted pathogenesis of thyrotoxicosis and various pharmacological treatment options, including beta-blockers and anti-thyroid drugs. This article has reviewed numerous studies that have concluded that the main goal of therapy should always aim to normalize thyroid hormone levels based on the etiology of the thyrotoxicosis, although cardiovascular conditions are associated with a higher rate of mortality.

## Introduction and background

Hyperthyroidism is a disorder that is defined as the increased production and release of thyroid hormone by the thyroid gland, which results in excessively high levels in the serum [1]. Thyrotoxicosis is a hyperdynamic and hypermetabolic syndrome that results in a multisystemic web of clinical manifestations, all of which stem from an inappropriately high level of circulating thyroid hormone in the body [1]. The most common causes of thyrotoxicosis include diffuse toxic goiter (Graves’ disease), toxic multinodular goiter (Plummer disease), and toxic adenoma [2]. Other causes include inflammation (silent thyroiditis) and medications like amiodarone and iodine [3]. According to recent statistics, the prevalence of hyperthyroidism was estimated to be around approximately 0.8% in Europe and 1.3% in the United States [4,5]. The incidence of hyperthyroidism increases with age, despite a minor propensity toward females compared to males; in addition, more Caucasians than other races are being affected [4]. Thyrotoxicosis can present with a spectrum of symptomatology ranging from heat intolerance, weight loss, palpitations, abnormal uterine bleeding to a life-threatening emergency known as thyroid storm [3]. Thyrotoxicosis can also manifest with organ decompensation leading to multisystem organ failure, commonly including the heart, lungs, liver, and kidneys [6]. While thyrotropin receptor antibodies, radioactive iodine scintigraphy, or thyroid blood flow via ultrasonography can all be used to diagnose thyrotoxicosis, the diagnostic accuracy improves when thyroid-stimulating hormone (TSH), free thyroxine (FT4), and free triiodothyronine (FT3) are also evaluated [7,8]. The management of hyperthyroidism is usually tailored around the patient profile and the cause of the disorder with options including drugs, radioactive iodine, and surgery [9]. In patients with symptomatic thyrotoxicosis with resting heart rates above 90 beats per minute or coexisting cardiovascular disease, the beta-adrenergic blockade is recommended as the first-line treatment. At the same time, anti-thyroid drugs, like carbimazole, propylthiouracil (PTU), and methimazole (MMI), can also be used; they are associated with side effects like agranulocytosis, vasculitis, or hepatic damage [8,10]. Physiologically, the thyroid hormone exhibits a close harmony with the cardiovascular system in maintaining systolic and diastolic blood pressure, heart rate, contractility, cardiac output, systemic vascular resistance, etc. [7,11]. Consequently, an increase in thyroid hormone directly affects the heart, leading to disturbances in hemodynamic stability and thus exacerbating the possibility of developing complications like heart failure (HF) and life-threatening arrhythmias [12,13]. In such clinical scenarios, although the incidence is minimal, the mortality rate of the patient is magnified severalfold. In this review article, we underlined the alterations in the hemodynamic parameters caused by thyroid hormones, explored the relationship between thyrotoxicosis and HF from a clinical perspective, and highlighted the screening and diagnostic guidelines in addition to the therapeutic management options for HF in thyrotoxicosis.

Methodology

Sample Population

Studies with both male and female populations, and of all geographical and ethnic backgrounds were included. We did not define any specific age groups.

Search Strategy and Inclusion and Exclusion Criteria

PubMed was the only database that was accessed, specifically between the years 2000 and 2021. The terminology mentioned in Table 1 was used to search the PubMed database.

**Table 1:** Terminology Used on PubMed

Terminology Used	Number of Results
Hyperthyroidism AND heart failure	581
Thyrotoxicosis AND heart failure	256
Thyrotoxicosis AND atrial fibrillation	246
Thyroid AND heart failure	832

**Figure 1:**
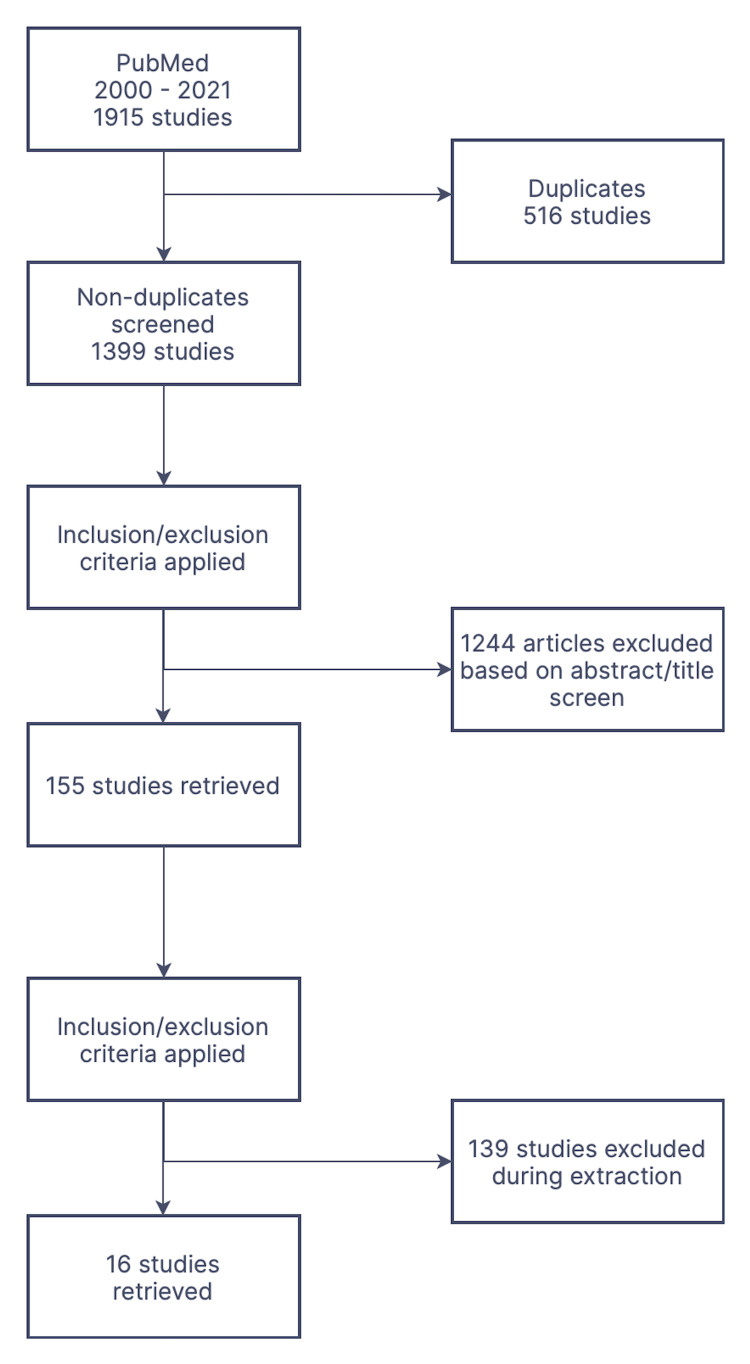
PubMed Data Extraction Using Inclusion and Exclusion Criteria

All types of studies were included including randomized controlled trials, cohort studies, case-control studies, comparative studies, and qualitative studies. Only human studies from peer-reviewed journals were included. No grey literature was included. The data extraction criteria applied are explained in Figure 1.

## Review

Hemodynamic changes in thyrotoxicosis

Thyroid hormone regulation is maintained via a delicate system involving the hypothalamus, pituitary gland, and thyroid gland. The hypothalamus releases thyroid-releasing hormone (TRH), which stimulates thyroid stimulation hormone release from the pituitary, which in turn stimulates the release of T3 and T4 from the thyroid gland [14]. In the thyroid gland, the production of thyroid hormones begins with iodine binding to thyroglobulin with the help of thyroid peroxidase, which then forms monoiodotyrosine (MIT) and diiodotyrosine (DIT), and consequently coupling to create T4 and T3 [15]. In the serum, the thyroid hormone is inactive as it is typically bound to protein (thyroglobulin). Any physiologic or pathologic process that can increase the amount of unbound thyroid hormone can cause thyrotoxicosis [14]. However, under ideal conditions, an excessive increase in the production of thyroid hormone initiates the negative feedback loop, which inhibits TRH released from the hypothalamus in addition to TSH release from the pituitary gland [14]. In pathologic conditions, this protective mechanism is not enough to negate the effects of the offending pathology.

The thyroid hormone is fundamental for normal development, growth, neural differentiation, and, most importantly, metabolic regulation [16]. Many cardiac functions such as heart rate, cardiac output, and systemic vascular resistance are affected by the functioning of the thyroid gland [13]. T3 is derived from T4 and is more biologically active [14]. T3 and T4 increase the resting cardiac output, stroke volume, and myocardial oxygen demand while decreasing peripheral vascular resistance, systemic vascular resistance, and smooth muscle contractility [17-19]. These hormones will also widen the pulse pressure due to increased systolic contractile function and diastolic relaxation [20]. The primary hemodynamic changes are summarized in Table 2.

**Table 2:** Hemodynamic Changes in Thyrotoxicosis

Hemodynamic Parameters	Changes
Blood pressure	Increase
Blood volume	Increase
Heart rate	Increase
Cardiac output	Increase
Cardiac contractility	Increase
Systemic vascular resistance	Decrease
Myocardial oxygen demand	Increase

The intracellular cardiac effects of the thyroid hormone are carried out by genomic mechanisms and non-genomic mechanisms, which regulate cardiac function and cardiovascular hemodynamics [13,21]. The genomic action involves T3 linking to nuclear receptors that bind to thyroid-responsive elements (TREs) in target genes [13]. This upregulates alpha-myosin heavy chain gene expression, sarcoplasmic reticulum calcium ATPase, sodium-potassium-ATPase, beta-1 adrenergic receptor, and voltage-gated potassium channels; all of which can increase the synthesis of cardiac proteins, leading to cardiac hypertrophy and eventual dysfunction [22]. The TREs also downregulate beta myosin heavy chain, phospholamban, adenylyl cyclase catalytic subunits, sodium-calcium exchanger, and thyroid hormone alpha-1 receptor, which causes inhibition of myocardial relaxation [22]. The non-genomic action involves rapid changes in the cardiac myocyte plasma membrane and cytoplasmic organelles, including sodium, potassium, and calcium ion channels, polymerization of the actin cytoskeleton, and intracellular signaling pathways not only in the heart but also the smooth muscle cells [21].

In thyrotoxicosis, the upregulated beta-adrenergic receptor results in increased tissue sensitivity to catecholamines, contributing to the symptoms of increased heart rate, blood volume, stroke volume, myocardial contractility, and ejection fraction [21]. Persistent levels of elevated thyroid hormone also increase the preload while reducing the peripheral vascular resistance (PVR), which contributes to an increased cardiac output [13]. The reduced PVR causes a decreased renal perfusion pressure leading to the activation of the renin-angiotensin-aldosterone system (RAAS) [21]. This increases sodium reabsorption and blood volume, increasing the preload and decreasing the afterload [21]. T3 is also evidenced to directly stimulate renin synthesis from the liver, leading to increased intracardiac levels of renin and angiotensin II independent of the circulating renin and angiotensin [13]. The hyperthyroid state increases myocardial expression of angiotensin II receptors, which causes atrial stretch, leading to the production of atrial natriuretic peptide (ANP), allowing more vasodilation, contributing to myocardial hypertrophy [21,22].

Cardiovascular involvement in thyrotoxicosis

Thyrotoxicosis is a clinical syndrome with persistent high concentrations of FT3, FT4, or both, which correlates with either increased or decreased thyroid metabolic function [23]. Thyroid storm, also referred to as thyrotoxic crisis, constitutes the severe end of the spectrum of thyrotoxicosis, which is frequently characterized by organ dysfunction and failure [24]. Thyrotoxicosis most commonly arises from Graves disease but can also be caused by toxic multinodular goiter, toxic adenoma, TSH-producing adenoma, or pituitary adenoma, human chorionic gonadotropin (HCG)-mediated hyperthyroidism, thyroiditis, factitious hyperthyroidism, iodine contrast, or medications like amiodarone [25]. Despite the absence of defining characteristics, certain clinical features that are uncommon in thyrotoxicosis can be used as major indicators for thyroid storm. This includes goiter, ophthalmopathy, hyperpyrexia, severe tachycardia, agitation, delirium, diarrhea, and jaundice [26]. The complications of thyroid storm can vary extensively due to the various effects of the thyroid hormones. Some complications are high-output HF, congestive HF, ventricular arrhythmias, atrial fibrillation, diarrhea, delirium, seizures, coma, dehydration, and elevated liver transaminases [26-29].

As per the latest literature, it was additionally found that patients with no significant past medical history were at an increased risk for HF due to concurrent cardiovascular conditions (particularly atrial fibrillation) caused by thyrotoxicosis [30]. A meta-analysis conducted by Larsson et al. observed the association with alterations in cardiac hemodynamics, including atrial fibrillation, coronary artery disease, and ischemic stroke in 72,167 individuals with decreased TSH levels, indicative of subclinical thyroid dysfunction. This study concluded that there was a significant association between decreased TSH levels and an increased risk of atrial fibrillation, but no other cardiovascular diseases (Table 3) [31]. A study conducted by Teasdale et al. monitored cardiac function using modern echocardiographic techniques in eight patients with Graves’ hyperthyroidism. It was concluded that Graves’ hyperthyroidism causes increased cardiac output and a hyperdynamic right ventricle as indicated by peak systolic velocity of the free wall of the tricuspid annulus, tricuspid annular plane systolic excursion, and right ventricular ejection fraction (Table 3) [32]. Yiu et al. conducted a study observing the clinical characteristics and major adverse cardiovascular events (MACEs), including cardiovascular mortality, myocardial infarction, stroke, and HF, or ventricular arrhythmias, in amiodarone-induced thyrotoxicosis. It was concluded that amiodarone-included thyrotoxicosis is associated with a left ventricular ejection fraction less than 45% and a 2.7-fold increased risk of MACEs (Table 3) [33]. A study conducted by Wustmann et al. aimed to determine the activity of abnormal supraventricular electrical depolarizations in 28 patients newly diagnosed and untreated hyperthyroidism without any previous history of structural heart disease. This study concluded that hyperthyroidism is strongly correlated with increased supraventricular ectopic activity associated with atrial fibrillation (Table 3) [34]. Sokmen et al. conducted a study to evaluate the diastolic function and atrial electromechanical delay, which commonly leads to atrial fibrillation in subclinical and overt hyperthyroidism using tissue Doppler imaging. It was concluded that intra-atrial and inter-atrial electromechanical intervals were prolonged, and the diastolic function was impaired in overt and subclinical hyperthyroidism (Table 3) [35]. A study conducted over 3.2 years aimed to determine the influence of subclinical hyperthyroidism and the risk of HF and cardiovascular diseases in older people between the ages of 70 and 82. After monitoring TSH and FT4 levels, it was concluded that older people with underlying cardiovascular risk factors with low TSH are at an increased risk of developing incidental HF (Table 3) [36]. Another study conducted over a period of 9 years in Copenhagen, Denmark, aimed to examine the risk factors related to mortality and MACEs with overt and subclinical thyroid dysfunction. After observing that the risk of MACEs was elevated in overt and subclinical hyperthyroidism leading to HF, it concluded that HF is the leading cause of increased cardiovascular mortality (Table 3) [37]. Mitchell et al. conducted a study over a period of 5 years to examine whether patients with systolic HF and abnormal thyroid function carry a greater risk of mortality. After careful monitoring of TSH levels every 6 months, it was concluded that abnormal thyroid function in patients with symptomatic systolic HF and decreased ejection fraction is closely related to an increased mortality rate, even after interventions such as amiodarone and implantable cardioverter-defibrillator therapy are applied (Table 3) [38]. As explained in the studies mentioned above, Table 3 explains the implications that thyrotoxicosis has on cardiovascular disease.

**Table 3:** The Correlation Between Thyrotoxicosis and Cardiovascular Disease TSH: thyroid-stimulating hormone; RV: right ventricle; HF: heart failure; EMD: electromechanical delay; FT4: free thyroxine; MACEs: major adverse cardiovascular events; SVPD: supraventricular depolarizations; SVT: supraventricular tachycardia

References	Design	Cases	Diagnostic Criteria	Conclusion
Larsson et al. (2019) [31]	Meta-analysis	72,167	TSH levels	There is an association between decreased TSH levels and an increased risk of atrial fibrillation.
Teasdale et al. (2017) [32]	Clinical Trial	8	Graves’ hyperthyroidism with intervention.	Graves’ hyperthyroidism caused an increased cardiac output and a hyperdynamic RV.
Mitchell et al. (2013) [38]	Randomized controlled clinical trial	2,225	TSH levels	Abnormal thyroid function in patients with symptomatic HF and an ejection fraction ≤35% can be correlated with an increased risk for death, even with interventions.
Sokmen et al. (2013) [35]	Controlled clinical trial	93	Atrial EMD and diastolic function	Electromechanical intervals were prolonged and diastolic function was impaired in both overt and subclinical hyperthyroidism.
Nanchen et al. (2012) [36]	Prospective cohort study	270	TSH levels	People aged 70 to 82 with low TSH and normal FT4 appear to be at an increased risk of HF.
Yiu et al. (2009) [33]	Retrospective cohort study	354	Baseline clinical characteristics, laboratory parameters, and outcome events were evaluated.	Amiodarone-induced thyrotoxicosis is associated with a 2.7-fold increased risk of MACEs.
Selmer et al. (2014) [37]	Retrospective cohort study	563,700	TSH levels	HF is the primary cause of cardiovascular-related mortality in both overt and subclinical hyperthyroidism.
Wustmann et al. (2008) [34]		28	Abnormal SVPD, number of episodes of SVT, heart rate oscillations, and heart rate variability.	Hyperthyroidism is strongly correlated with increased supraventricular ectopic activity associated with atrial fibrillation.

Diagnosis

In symptomatic thyrotoxicosis, serum TSH is decreased while free thyroxine (T4) or free thyroxine and free triiodothyronine (T3) are increased; however, subclinical thyrotoxicosis presents with persistently low serum concentration of TSH, with normal concentrations of free T3 and T4 [39]. In some cases, the thyroid radioiodine uptake and scan may be used to help identify the underlying etiology [39]. Using the initial diagnosis of thyrotoxicosis as a prerequisite, Akamizu et al. developed specific criteria to diagnose thyrotoxicosis that looked at diagnostic criteria in addition to incidence, clinical features, mortality, and prevalence of survival with complications [40,41]. The authors developed diagnostic criteria for thyrotoxicosis based on seven of their patients and 99 patients from the literature [41]. This included symptoms of restlessness, delirium, mental aberration/psychosis, somnolence/lethargy, convulsion, or coma, a fever of 38°C or higher, tachycardia, which consisted of a heart rate greater than 130 beats per minute [39]. Other symptoms included congestive HF with pulmonary edema, moist rales, or cardiogenic shock in addition to gastrointestinal and hepatic manifestations such as nausea, vomiting, diarrhea, or jaundice [40-41].

As the thyroid hormone naturally affects the regular functioning of the heart, an increase in thyroid hormone may complicate pre-existing cardiac dysfunction or cause severe cardiac complications in individuals with functionally healthy hearts [42]. Most commonly, rhythm disturbances like atrial fibrillation or right bundle branch block, high cardiac output HF, cardiomyopathy, and systolic hypertension are observed [11,43]. HF due to thyrotoxicosis is clinically suspected by the presence of any of the following symptoms including, tachycardia, palpitations, wide pulse pressure, hyperactive precordium, dyspnea on exertion, or generalized fatigue [43]. Other signs may include diminished myocardial contractility, decreased systemic vascular resistance, increased preload, increased cardiac output, and increased blood volume [43]. To diagnose HF in the presence of underlying thyrotoxicosis, the serum levels of FT4, FT3, TSH should be considered first [44]. Ultrasound thyroid scan, Doppler imaging of the thyroid, standard 12-lead electrocardiogram (ECG), 24-hour Holter ECG, and complete Doppler echocardiography of the heart can also monitor heart activity, which would be abnormal or increased in the presence of underlying thyrotoxicosis [44,45].

Treatment of heart failure in thyrotoxicosis

HF secondary to thyrotoxicosis is predominantly resolved by lowering peripheral thyroid hormone levels, which aids in the reversal of systemic decompensation [44]. The main goals of treatment and management of thyrotoxicosis are to reduce circulating thyroid hormone levels and block peripheral effects of circulating thyroid hormone [44]. Gazzana et al. conducted a study to evaluate the effects of hyperthyroidism and the possibility of reversing the effects on cardiovascular structure and function using Doppler echocardiography. It was concluded that patients with hyperthyroidism developed cardiovascular changes, increased cardiac chamber size, cardiac output, left ventricular ejection fraction (LVEF), and pulmonary artery systolic pressure. These changes were evidenced to be reversible after lowering the levels of FT4 back to normal in patients without the pre-existing cardiovascular disease (Table 4) [46].

Additionally, Shuvy et al. conducted a study observing the heart rate variability (HRV) in thyroxine suppressive therapy, as HRV is a sensitive marker of cardiac sympathetic activity. The 1-minute HRV was calculated from the difference in beats per minute between the shortest and the longest heart rate interval during six cycles of deep breathing measured by electrocardiography. Results showed that the 1-minute HRV was significantly lower in thyroxine-treated patients than healthy controls, concluding that thyroxine suppressive therapy decreases HRV by way of autonomic dysregulation (Table 4) [47]. Another study conducted by Tomisti et al. over a period of 3 years at the University of Pisa observed the effect of a total thyroidectomy on cardiac function and overall survival of patients with amiodarone-induced thyrotoxicosis with severe left ventricular systolic dysfunction. After undergoing thyroidectomy and receiving levothyroxine replacement therapy, the LVEF improved in patients with LV systolic dysfunction. It was concluded that by restoring euthyroidism, cardiac function and the risk of mortality are significantly diminished (Table 4) [48].

Blocking the synthesis of thyroid hormone is the action of agents known as the thionamides, also known as anti-thyroid drugs, including PTU and MMI [49]. These drugs are commonly associated with liver dysfunction, but PTU is the first-line drug used for hyperthyroidism, as it has the least risk of hepatotoxicity [50]. As thyrotoxicosis is primarily seen in middle-aged women, it is essential to know that PTU is also recommended in the first trimester of pregnancy due to decreased teratogenic effects over MMI, which is used during the second trimester of pregnancy [50]. In a study conducted by Takata et al. over 5 years in a sample population of 134 untreated patients with Graves’ disease, who compared the effect of MMI treatment with MMI and potassium iodide (KI) treatment in rapid normalization of thyroid hormones during the early phase of thyrotoxicosis. They also monitored disease remission after 5 years. It was concluded that combined therapy with MMI and KI improved the short-term control of Graves’ hyperthyroidism by normalizing FT3 levels and was not associated with worsening hyperthyroidism or drug resistance (Table 4) [51].

Another option is to block to release of preformed thyroid hormone using lithium carbonate or inorganic iodine components, like Lugol’s solution or potassium iodide [49]. These are commonly used in combination with beta-adrenergic blockers, specifically propranolol, for significant improvement in thyroid hormone levels [52]. To block the effects of thyroid hormone against peripheral tissues, specifically the hyperadrenergic symptoms, beta-blockade using propranolol or esmolol can be used [49]. Palmieri et al. conducted a study observing the effects of acute beta-1 adrenergic blockade (bisoprolol) on myocardial contractility and total arterial stiffness in patients with thyrotoxicosis. It was observed that in a hyperthyroid state, there is a sustained increase in preload with enhanced LV diastolic function. In patients treated with bisoprolol, there was decreased cardiovascular hyperkinesia, which manifested as a lowered heart rate. It was concluded that specific beta-1 adrenergic blockade using bisoprolol leads to the normalization of total arterial stiffness, which attenuates the high-output state commonly seen in thyrotoxicosis patients (Table 4) [53]. Beta-blockade can be used as sole therapy to provide symptomatic relief in the short term [54]. However, beta-blockers are used in combination with radioactive iodine or anti-thyroid drugs for long-term treatment [54]. Tagami et al. conducted a study over a period of 1 month with beta-blockers in a sample population of 28 adults to observe its effects on new-onset thyrotoxicosis caused by Graves’ disease. It was found that symptoms of shortness of breath and fatigability in addition to heart rate all improved with adjunctive beta-blocker therapy than with MMI therapy alone (Table 4) [55].

Peripheral conversion from T4 to T3 can be inhibited using PTU, propranolol, glucocorticoids such as dexamethasone or hydrocortisone, or oral contrast agents like iopanoic acid [49]. Glucocorticoid administration results in inhibition of TSH release, which allows the thyroid hormone level to reduce, controlling the symptoms of thyrotoxicosis [56]. An exploratory study conducted with a sample population of three patients was observed for seven days for the effects of high-dose IV glucocorticoids compared to standard-dose oral glucocorticoids in amiodarone-induced thyrotoxicosis. It was concluded that high-dose IV glucocorticoid therapy does not offer advantages over standard-dose oral glucocorticoid therapy in the rapid, short-term period (Table 4) [57]. Rarely, medications like lithium, hemodialysis, charcoal hemoperfusion, and cholestyramine can also be used to treat symptoms of thyrotoxicosis [58]. Hemodialysis and charcoal hemoperfusion clarify the blood by increasing the excretion of thyroid hormone [58]. Cholestyramine is effective because it will bind the thyroid hormone, usually reabsorbed in the distal small intestine, reducing the effective amount of circulating thyroid hormone [59]. A study conducted by Kaykhaei et al. studied the effects of low-dose cholestyramine on serum total triiodothyronine and free thyroxine. They concluded that cholestyramine when compared to methimazole and propranolol, is more effective in decreasing serum levels of thyroid hormones (Table 4) [60]. Another study conducted over a period of 5 weeks in a sample population of 15 patients with thyrotoxicosis observed the effects of cholestyramine, an anion exchange resin that binds iodothyronines, in adjunction with thionamides and atenolol, a beta-blocker. After weekly monitoring FT4, FT3, TSH, and thyrotropin-binding inhibitory immunoglobulin, it was concluded that cholestyramine is most effective in treating thyrotoxicosis during the first few weeks of treatment [59]. Last, prevention against systemic decompensation can be done via acetaminophen to prevent hyperthermia and dextrose and electrolytes to prevent dehydration [46]. Hyperthyroidism has a significant clinical impact as it affects cardiac morphology and function. Therefore, appropriate treatment and intervention should be considered to attenuate specific symptoms and signs of elevated thyroid hormone and avoid the permanent cardiac complication of long-term exposure to excess thyroid hormone [45]. As mentioned in the studies above, the various treatment regimens for HF secondary to thyrotoxicosis are illustrated in Table 4.

**Table 4:** Treatment of Heart Failure Secondary to Thyrotoxicosis FT3: free triiodothyronine; FT4: free thyroxine; TSH: thyroid-stimulating hormone; IV: intravenous; RV: right ventricle; MMI: methimazole; KI: potassium iodide; LV: left ventricle

References	Design	Cases	Diagnostic Criteria	Conclusion
Shuvy et al. (2008) [47]	Controlled clinical trial	38	FT4, FT3, and TSH levels. One-minute heart rate variability.	Thyroxine suppressive therapy resulted in subclinical hyperthyroidism and significantly decreased heart rate variability due to autonomic dysfunction.
Cappellani et al. (2020) [57]	Exploratory study/clinical trial	12	Serum thyroid hormone concentrations	High-dose IV glucocorticoid therapy does not offer advantages over standard oral glucocorticoid therapy.
Gazzana et al. (2019) [46]	Prospective cohort study	32	FT4 and echocardiogram. Exclusion criteria included previous cardiovascular disease.	Increased cardiac chambers, cardiac output, and impaired RV function were observed in hyperthyroid patients, which were reversible after FT4 normalization.
Tagami et al. (2012) [55]	Randomized controlled trial	28	Elevated heart rate and thyroid function, compromised quality of life.	Adjunctive beta-blocker therapy is more effective than MMI alone.
Tomisti et al. (2012) [48]	Retrospective cohort study	39	Left ventricular ejection fraction	Total thyroidectomy may improve cardiac function and reduce the risk of mortality in amiodarone-induced thyrotoxicosis patients with severe left ventricular dysfunction.
Takata et al. (2010) [51]	Randomized controlled trial	134	Serum FT4, FT3, TSH, and TSH receptor antibodies. Goiter size	Combined treatment with MMI and KI improved the short-term control of Graves’ hyperthyroidism.
Kaykhaei et al. (2008) [60]	Prospective, randomized, double-blind, placebo-controlled trial	45	Serum T3 and T4	Low-dose cholestyramine is effective in decreasing serum thyroid hormone levels.
Palmieri et al. (2004) [53]	Clinical trial	30	LV structure and function, hemodynamics, and total arterial stiffness.	Beta-1 adrenergic blockade leads to decreased cardiovascular hyperkinesia, heart rate, and total arterial stiffness.

Limitations

This study did not take the genetic predisposition or the environmental factors of patients with hyperthyroidism or thyrotoxicosis into consideration, which can potentially serve as confounding variables. Additionally, the cardiac conditions that have been reviewed in this article have a multitude of etiological origins and usually involve more than a single causative factor, such as thyrotoxicosis. This study did not take any bias into consideration.

## Conclusions

From the studies discussed in this article, thyrotoxicosis comprises a wide spectrum of clinical manifestations. As many metabolic processes involve the thyroid hormone, thyrotoxicosis can contribute to multisystem dysfunction and eventual failure primarily involving the heart, lungs, liver, and kidneys. Since the cardiovascular system is the most commonly affected, careful observation should be done to avoid misdiagnosing symptoms like a third heart sound or pulmonary congestion, which could lead to atrial fibrillation and HF. It is imperative to monitor HF with underlying thyrotoxicosis with the regular observation of TSH, T4, and T4 levels, to confirm normal values. In addition, antibody titers, radioactive iodine scintigraphy, USG, echocardiography, and tissue Doppler imaging can also be utilized. This decision should be made based on the presentation of the patient, who should be carefully examined while taking their age and comorbidities into consideration. Regular monitoring with repeat measurements can assist in avoiding the negative effects associated with overtreatment. The clinical implications of this article are to identify the significance of HF in thyrotoxicosis while reviewing the process of decision-making to treat HF secondary to hyperthyroidism or thyrotoxicosis. Furthermore, we also believe that there needs to be more comprehensive research concerning different pharmacological treatments used in thyrotoxicosis and their effects on cardiac remodeling.
